# Unveiling the links between physical activity, self-identity, social anxiety, and emotional eating among overweight and obese young adults

**DOI:** 10.3389/fpsyg.2023.1255548

**Published:** 2024-01-08

**Authors:** Huilin Wang, Xianyi He, Yiwei Tang, Jiaxin Tang, Jingyu Yang

**Affiliations:** ^1^School of Business, Hunan University of Science and Technology, Xiangtan, China; ^2^School of Physical Education, Hunan University of Science and Technology, Xiangtan, China; ^3^College of Physical Education, Hunan Normal University, Changsha, China; ^4^Department of Medical Bioinformatics, University of Göttingen, Göttingen, Germany

**Keywords:** physical activity, emotional eating, overweight and obese young adults, social anxiety, self-identity

## Abstract

**Introduction:**

Emotional eating not only contributes to physical obesity but also leads to the experience of guilt and shame, exacerbating emotional problems. Increasing physical activity, adopting a balanced diet, and seeking psychological support help improve emotional eating issues in overweight or obese young adults, enhancing overall mental and physical well-being.

**Methods:**

This study investigates the correlation between physical activity, self-identity, social anxiety, and emotional eating among 373 overweight and obese college students aged 18–26 in central China. By utilizing AMOS v.26, a structural equation model was constructed to examine the hypotheses.

**Results:**

The findings reveal that physical activity significantly influences self-identity and social anxiety, which, in turn, significantly impact emotional eating. Moreover, self-identity and social anxiety serve as mediators in the relationship between physical activity and emotional eating. These results emphasize the role of physical activity in mitigating emotional eating among young individuals struggling with overweight and obesity.

**Discussion:**

Consequently, the government and relevant agencies are urged to address the issue of obesity among young adults and provide support for their engagement in physical activity.

## 1 Introduction

According to the 2020 Report on Chinese Nutrition and Chronic Disease (State Council Information Office, 2020), the prevalence of overweight and obesity among Chinese adults stands at 34.3 and 16.4%, respectively. Furthermore, major chronic diseases account for a significant proportion of premature mortality, with a mortality rate of 16.5% and chronic diseases responsible for 86.6% of total deaths. Additionally, there has been a notable rise in obesity-related and diet-related chronic conditions such as hypertension, cardiovascular disease, and type 2 diabetes in recent years (Wang et al., 2007). Sedentary lifestyles and reduced physical activity contribute to the increased risk of obesity and various chronic conditions. The economic impact of obesity on a global scale is overwhelming, amounting to approximately 2 trillion USD or 2.8% of the worldwide GDP, rivaling the impact of smoking or armed conflicts (Dobbs et al., 2014). As pointed out by Malik and Hu (2022), obesity represents one of the most pressing public health challenges in the 21st century.

Emotional eating refers to the tendency to overeat in response to stress and negative emotions, such as anger, fear, boredom, sadness, and loneliness, as a means of coping with emotional fluctuations (Risica et al., 2021). It is more prevalent among individuals who are overweight or obese (Betancourt-Núñez et al., 2022). Significant life events or everyday frustrations can trigger negative emotions, leading individuals to seek solace in food, consciously or unconsciously, thereby sabotaging their weight loss efforts (Cardoso et al., 2020; Ljubičić et al., 2023). Moreover, stress and anxiety compel individuals to increase their energy intake, with high-sugar foods promoting serotonin production and positively impacting mood (Madalı et al., 2021). Regardless of the underlying reasons that drive overweight and obese young people to engage in binge eating, the comforting effects derived from these food choices are temporary (Bennett et al., 2013). Consequently, when the emotional surge subsides, overweight and obese young adults may experience additional guilt regarding their failed attempts at weight loss (Hawks et al., 2008). This perpetuates a vicious cycle wherein individuals resort to overeating due to emotional fluctuations, subsequently berating and shaming themselves for their eating behavior, ultimately leading to feelings of depression and further overeating. Consequently, their weight spirals out of control within this relentless cycle (Wong and Qian, 2016).

Previous research on emotional eating among overweight and obese individuals has primarily focused on factors such as depression (Bennett et al., 2013), anxiety (Fox et al., 2016), stress (Diggins et al., 2015), quality of life (Giusti et al., 2022), and negative body image (Shriver et al., 2020). However, in contrast to these studies, the researchers posit that depression, anxiety, stress, and negative body image merely represent emotional and behavioral manifestations. The underlying cause lies in the low self-identity prevalent among overweight and obese young people. When faced with external stimuli such as unfamiliar environments, challenges, and rejection from others, individuals with lower self-identity are more susceptible to negative emotions. Additionally, overweight and obese young people exhibit higher levels of social anxiety, fearing discrimination in their daily social interactions and, thus, becoming more inclined to withdraw from social engagements. This poses long-term risks in terms of sedentary behavior and physical inactivity.

However, the researchers contend that the collective nature of physical activity holds the key to unlocking the potential within overweight and obese young adults. Engaging in physical activity helps them establish a robust knowledge system and cognitive framework concerning health and body image, while also providing opportunities for social interaction that enable them to perceive themselves accurately and overcome the fear of external judgment. Furthermore, physical activity aids overweight and obese young adults in breaking free from the aforementioned vicious cycle, fostering a regular and self-disciplined lifestyle, and empowering them to master their own emotions. Based on the aforementioned research gaps and questions, this study aims to achieve the following objectives: (1) comprehend emotional eating patterns among overweight and obese young people in China, (2) explore the interrelationship between physical activity, self-identity, social anxiety, and emotional eating, and (3) identify existing issues and provide recommendations to relevant stakeholders.

This study addresses the issue of emotional eating among overweight and obese young individuals, proposing that physical activity can effectively mitigate emotional eating behaviors. The specific pathways are as follows: physical activity assists overweight and obese young people in cultivating a positive sense of self-identity, enabling them to overcome low self-esteem and self-denial in the face of challenges; the social aspects of physical activity enhance social opportunities for overweight and obese young individuals, and cooperative exercise programs help alleviate social anxiety; engaging in social activities aids in fostering positive emotions among overweight and obese young people, thereby improving their habits of overeating triggered by mood swings and uneasiness. The study’s findings indicate the critical role of self-identity in the development of social anxiety. Furthermore, self-identity and social anxiety mediate the relationship between physical activity and emotional eating, implying that physically active overweight and obese young individuals possess stronger self-identity and lower levels of social anxiety, resulting in reduced emotional eating behaviors. Therefore, understanding the impact of physical activity on emotional eating behaviors among overweight and obese young individuals not only contributes to the advancement of interdisciplinary theories but also underscores the importance of addressing the physical and mental health issues faced by this population. Moreover, it calls for the provision of greater convenience and support, from the government, schools, and families, for the participation of overweight and obese young individuals in physical activities.

As dietary patterns shift and sedentary behaviors become more prevalent, obesity has become a widespread concern across all age groups (Hoare et al., 2016), including children, young adults, and the elderly (Strauss et al., 2001; Wuthrich et al., 2019; Carraça et al., 2021). However, obesity tends to be particularly prevalent among young individuals. Apart from the physical health implications, obesity can have severe mental health consequences (Jepsen et al., 2014), manifesting as social anxiety, diminished self-identity, increased stress, and psychological distress (Carraça et al., 2021). Encouraging evidence suggests that physical activity plays a crucial role in instilling confidence, fostering positive relationships, and bolstering self-identity through sports and recreational activities (Whatnall et al., 2021). Furthermore, research indicates that physical activity serves as an effective intervention to address psychological issues in children, including anxiety and depression, which often accompany obesity (Karampatsou et al., 2022). In the case of obese women, physical activity not only aids in weight loss but also facilitates communication and camaraderie within exercise groups, thereby mitigating feelings of inferiority, psychological distress, and social anxiety associated with obesity (Ekkekakis et al., 2010).

Self-identity manifests in various forms, primarily through individual sports and cultural affiliations (Yoon et al., 2013). It is a fundamental need for individuals (Dien, 2000), and it is strongly correlated with intentions in the theory of planned behavior, significantly impacting overall well-being and self-efficacy (Brown et al., 2012; Ries et al., 2012). Individuals with a strong sense of self-identity are less likely to experience social anxiety and loneliness, leading to enhanced self-awareness and self-understanding (Pallesen, 2014). Social anxiety, characterized by fear of social interactions and lack of self-confidence, is often prevalent among modern young individuals struggling with obesity, highlighting the need for bolstering self-identity to alleviate social anxiety (Etkin and Wager, 2007). Obese young individuals often grapple with anxiety and low self-esteem in their professional and personal lives. Prolonged social pressures exacerbate emotional imbalances, prompting them to resort to emotional eating as a coping mechanism for emotional highs and lows (Brosof et al., 2019). Moreover, self-identity exerts a significant influence on emotions. For instance, previous studies have established a behavioral link between emotional eating and anxiety (Ostrovsky et al., 2013). Furthermore, scholars have highlighted the role of self-identity in providing emotional support, which contributes to alleviating anxiety and feelings of loneliness in personal and professional contexts, reducing emotional eating behaviors, promoting positive changes in dietary habits, and improving mental well-being (Dressler and Smith, 2013).

Existing studies examining the mediating role of self-identity and social anxiety have primarily focused on areas such as social support (Li et al., 2022), social environment (Wen et al., 2021), acculturation and needs (Yoon et al., 2013), and mental health issues (Zhou and Cheng, 2022). Additionally, some studies have identified the relationship between physical activity and self-identity and personal emotions (Dressler and Smith, 2013). Engaging in physical activity has been shown to improve mood (Whatnall et al., 2021), enhance self-identity (Whatnall et al., 2021), and reduce social anxiety (Ekkekakis et al., 2010). Moreover, evidence suggests that individuals with higher levels of self-identity experience lower levels of social anxiety. Consequently, as social anxiety decreases, an individual’s emotional stability gradually improves, leading to more regulated eating behaviors (Zhou and Cheng, 2022).

## 2 Methods

### 2.1 Participants and procedure

This study employed a purposive sampling method in its quantitative research approach, utilizing a cross-sectional design and a paper questionnaire as the data collection method. The survey targeted overweight and obese college students from eight universities located in Central China. Well-trained research assistants were responsible for gathering data from college students aged 18–26 within these institutions, during the period from July to September 2022. It is important to note that permission to conduct the research was obtained from the relevant institutional authorities, ensuring ethical and procedural compliance (No. ECSPEHNUST 2022/0012).

The participants’ academic majors and grades varied. Research assistants distributed paper questionnaires to participants in various contexts, including after academic classes, during extracurricular activities, and in the evening study sessions. Prior to the survey, the researchers provided a brief introduction regarding the survey’s purpose and content, obtained informed consent from the participants, and assured them of the anonymity of the study. The survey results were strictly intended for academic research purposes. A total of 373 overweight or obese college students were recruited to complete the questionnaire, with 357 valid questionnaires ultimately being returned, resulting in a valid questionnaire recovery rate of 95.7%.

The sample consisted of college students, with 78.2% being male and 21.8% female, and a mean age of 21.3 years. Among them, 60.8% of the respondents were majoring in science and engineering, and 77.3% were classified as sophomores and juniors. Furthermore, 73.1% of the participants were classified as overweight, while 26.9% fell into the obese category, as defined by the WHO Expert Consultation for Asians (WHO Expert Consultation, 2004).

### 2.2 Questionnaire

The questionnaire employed in this study consisted of five sections. The first section gathered demographic information from the respondents, including gender, age, major, grade, and degree of overweight or obesity. The second section assessed the respondents’ physical activity during the previous week using a five-item scale developed by Andersen et al. (2010) and Guillén and Martínez-Alvarado (2014). Sample items included statements such as “During the previous week, I frequently engaged in light physical activity.” The third section collected data on the respondents’ self-identity, measured using four items from the “Aspects of Identity Questionnaire III” developed by Cheek (1989). Sample items included statements such as “My sense of being a unique individual, distinct from others.” The fourth section gathered data on the respondents’ social anxiety, measured using four items from the “Liebowitz Social Anxiety Scale” developed by Heimberg et al. (1999). Sample items included statements such as “I experience anxiety when I act, perform, or speak in front of an audience.” Finally, the fifth section assessed emotional eating using four items from the “Emotional Eating Scale” developed by Arnow et al. (1995). Sample items included statements such as “When I feel lonely/sad/depressed, I tend to overeat.” All of the scales mentioned above were measured using a five-point Likert scale, ranging from 1 (strongly disagree) to 5 (strongly agree).

To ensure the appropriateness of the scale items within the Chinese cultural background and context, some expressions were revised accordingly. However, the researchers conducted a pilot test in two university classes to maintain the reliability of the revised scale. A total of 61 valid questionnaires were collected during the pilot test. The results indicated that the Cronbach’s coefficients for all four scales exceeded 0.8, indicating that the revised scale remained reliable and suitable for use in the formal survey.

### 2.3 Data analysis

This study employed AMOS v.26 for data analysis. To address common method variance (CMV), the researchers followed the approach recommended by Mossholder et al. (1998). They compared the degrees of freedom and chi-square values between model one and model two. The results indicated that the chi-square value for model 1 was 2361.517, with 77 degrees of freedom and a *p*-value less than 0.001. Similarly, the chi-square value for model 2 was 127.556, with 71 degrees of freedom and a *p*-value less than 0.001. These findings suggest that the fit of model 1 is equivalent to that of model 2. Therefore, the results indicate that there is no one-factor structure present, confirming that CMV is not a concern in this study.

## 3 Results

The structural model was analyzed using AMOS v.26 to test the hypotheses. The results of the confirmatory factor analysis (CFA) with 5,000 bootstrap samples demonstrated good fit indices (χ^2^/df = 2.52, GFI = 0.906, NFI = 0.952, CFI = 0.970, TLI = 0.965, RMSEA = 0.065), surpassing the recommended values by Hair, Black (Hair et al., 2010) (χ^2^/df < 3, GFI > 0.9, NFI > 0.9, CFI > 0.9, TLI > 0.9, RMSEA <0.08). This indicates a favorable fit of the model to the data.

**Figure 1:**
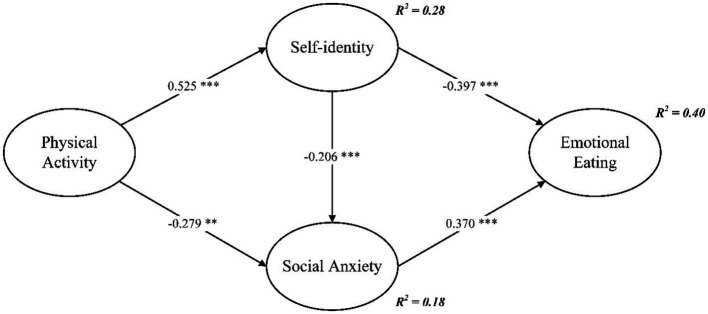
Structural path model; ** *p* < 0.01, *** *p* < 0.001. Standardized coefficients are reported.

Figure 1 illustrates the standardized coefficients for the variables in the structural equation model. As depicted in Figure 1, physical activity exhibited a direct and positive association with self-identity (*β* = 0.525, *p* < 0.001) and displayed a direct and negative association with social anxiety (*β* = −0.279, *p* < 0.01). Furthermore, self-identity demonstrated a direct and negative association with social anxiety (*β* = −0.206, *p* < 0.001) and emotional eating (*β* = −0.397, *p* < 0.001). Additionally, social anxiety demonstrated a direct and positive association with emotional eating (*β* = 0.370, *p* < 0.001).

Table 1 presents the results of the bootstrap estimation with 5,000 resamples and 95% bias-corrected confidence intervals, which were used to assess the mediating effect (Bollen and Stine, 1990). The findings indicated that there was a significant indirect effect of physical activity on social anxiety, which was mediated by self-identity (indirect effect = −0.108, SE = 0.048, CI = [−0.212, −0.026], *p* < 0.01). Additionally, there was a significant indirect effect of self-identity on emotional eating, which was mediated by social anxiety (indirect effect = −0.076, SE = 0.032, CI = [−0.145, −0.020], *p* < 0.01). Furthermore, there was a significant indirect effect of physical activity on emotional eating, which was mediated by self-identity and social anxiety (indirect effect = −0.352, *SE* = 0.039, CI = [−0.428, −0.276], *p* < 0.001). These results suggest that overweight and obese individuals who engage in more physical activity, possess a stronger sense of self-identity, and experience lower levels of social anxiety are inclined to engage in less emotional eating.

**Table 1:** Standardized indirect effects.

	Point estimate	Product of coefficients		Bootstrapping		
				Bias-corrected 95% CI		Two-tailed significance
		SE	Z	Lower	Upper	
PA → SA	−0.108	0.048	2.250	−0.212	−0.026	< 0.01
SI → EE	−0.076	0.032	2.375	−0.145	−0.020	< 0.01
PA → EE	−0.352	0.039	−9.026	−0.428	−0.276	< 0.001

Standardized estimations of 5,000 bootstrap samples.

## 4 Discussion

### 4.1 Theoretical contribution

Firstly, this study makes a theoretical contribution to the field of overweight and obesity research. Previous studies have primarily focused on the association between overweight and obesity and chronic diseases as well as psychological issues (Clark and Brancati, 2000; Bener and Tewfik, 2006), along with evaluating the effectiveness of medical and psychological interventions (Asayama et al., 2003; Chu et al., 2019). While some studies have examined the relationship between emotional eating and obesity, it has been established that emotional eating is a contributing factor to adult obesity (Konttinen, 2020). However, there is a gap in current research regarding how to address emotional eating in overweight and obese populations from both physiological and psychological perspectives. The findings of this study highlight that physical activity indirectly influences emotional eating in overweight and obese individuals through its direct effects on self-identity and social anxiety. Thus, the researchers contend that physical activity can effectively mitigate emotional eating in overweight and obese populations.

Secondly, this study contributes to the theoretical research on emotional eating. It is the first to explore the link between self-identity and emotional eating, confirming that individuals with a stronger sense of self-identity exhibit less emotional eating behavior. The study emphasizes that overweight and obese young adults often experience significant mood fluctuations due to their low self-identity, making them susceptible to external influences and evaluations. Their self-awareness may be vague, and they are prone to self-denial and easily giving up when faced with challenges. By enhancing their self-identity and reducing sensitivity to external information, this group can effectively address emotional eating triggered by positive or negative moods.

Thirdly, this study enriches the theoretical research on physical activity. The positive impact of physical activity on individuals’ physical and mental well-being has been well-established (Bertheussen et al., 2011; Wang et al., 2023). According to the study results, engaging in physical activity also contributes to fostering a sense of self-identity and building resilience in individuals, enabling them to face adversity with confidence. Moreover, physical activity often involves group participation. When overweight and obese young adults participate in physical activities, they have more opportunities for social interaction and are less likely to experience feelings of isolation or exclusion from society. Regular exercise and social engagement can benefit this group by reducing anxiety, fostering a more joyful and outgoing demeanor, and facilitating improved communication skills.

### 4.2 Practical implications

Considering the significant and negative impact of self-identity on emotional eating in overweight and obese young adults, it becomes evident that those with a stronger sense of self-identity exhibit lower levels of emotional eating. Numerous studies have demonstrated that individuals who experience humiliation and discrimination due to their obesity are more likely to gain further weight (Sutin and Terracciano, 2013). This is particularly relevant for individuals with low self-identity, as unintentional remarks or actions from others can deeply affect them. Young adults, in their pursuit of intimacy and employment opportunities, are prone to self-denial and self-abandonment when their self-identity is lacking (Pearce et al., 2002). During challenging times, they often attribute their failures to their weight. Frequent emotional fluctuations and bouts of depression make them more vulnerable to emotional eating. Consuming large quantities of high-fat, high-sugar, and high-calorie foods triggers the release of dopamine in the brain, providing a sense of pleasure (Baugh et al., 2022). Therefore, addressing emotional eating in overweight and obese young adults begins by improving their self-identity, reducing their sensitivity to external stimuli, and fostering self-confidence.

Furthermore, social anxiety exerts a significant and positive influence on emotional eating, indicating that individuals with higher levels of social anxiety tend to engage in more severe emotional eating behaviors. Overweight and obese young people, due to their outward appearance, often experience feelings of inferiority and heightened sensitivity, fearing rejection and discrimination when interacting with others. Consequently, they tend to close themselves off emotionally, develop social fears (Kanellopoulou et al., 2022), or excessively seek approval in social interactions. The term “fat otaku,” originating from Japan and now popular in China (Tang and Li, 2022), refers to obese individuals who prefer staying at home, have limited social interactions, consume large quantities of food, and indulge in high-calorie snacks (Yueh, 2019). This population is growing in China, and their sedentary behavior and lack of physical activity increase their risk of developing chronic diseases over time. Conversely, overweight and obese young adults with lower levels of social anxiety tend to have more extensive social circles, exhibit greater sociability, and find it easier to communicate in social settings, thus experiencing fewer instances of emotional eating.

The above discussion underscores the importance of enhancing self-identity and reducing social anxiety in addressing emotional eating issues among overweight and obese adults. The results of this study indicate that physical activity contributes to increased self-identity and reduced social anxiety in this population. While physical activity may not directly impact emotional eating, it indirectly influences it by affecting self-identity and social anxiety. Encouraging overweight and obese adults to adopt a physically active lifestyle proves to be an effective way to address the issues of low self-identity, severe social anxiety, and frequent emotional eating. To address the unique needs of obese college students, educational institutions should not only provide comprehensive sports facilities but also take measures to help them master scientifically sound exercise methods, improving their body composition and physical form. For instance, incorporating aerobic exercise and resistance training as non-pharmacological interventions into the weight-loss training programs for college students can achieve desired obesity control outcomes. Additionally, it can contribute to enhancing overall physical fitness, instilling the concept of lifelong physical activity among this student population.

In conclusion, promoting a physically active lifestyle is instrumental in improving self-identity, reducing social anxiety, and ultimately addressing emotional eating issues among overweight and obese adults. For obese college students specifically, a holistic approach that includes suitable physical activities and educational support can contribute to both effective weight management and the cultivation of a lifelong commitment to physical fitness.

### 4.3 Limitations

This study has several limitations that need to be acknowledged. Firstly, the researchers did not account for the presence of underlying health issues, such as chronic diseases, among overweight and obese young adults. It is important for future research to thoroughly consider this aspect, as not all individuals in this population may be suitable for certain types of physical activity, and the recommended exercises may vary based on their specific health conditions. Secondly, as this study was conducted using a cross-sectional design, the researchers focused on promoting physical activity as a means to improve emotional eating in overweight and obese young adults. However, the issue of promoting self-discipline to maintain a consistent exercise routine was not extensively explored. It is recommended that future studies delve deeper into this aspect, employing longitudinal research methods and incorporating experimental controls to further investigate this research topic. Thirdly, the quantitative research methodology utilized in this study has inherent limitations in exploring the relationship between physical activity and emotional eating. While it can establish a basic correlation, it falls short of providing an in-depth exploration of the associations and uncovering additional relevant factors. It is advisable for subsequent research to consider employing qualitative research methods or a mixed-method approach, as these could offer a richer understanding of participants’ experiences and perspectives.

## 5 Conclusion

Regarding the research objectives, this study found that a significant proportion of overweight and obese young adults, approximately 40–50%, reported severe binge eating triggered by various emotions, while about 25–30% experienced occasional emotional eating. The findings further highlight the significance of physical activity, self-identity, and social anxiety as influential factors in emotional eating among over-weight and obese young adults. Specifically, the study reveals that physical activity’s impact on emotional eating is mediated by both self-identity and social anxiety. Consequently, the study suggests that governmental bodies and relevant organizations should prioritize addressing the issue of obesity among young individuals, intervening at an early stage, and offering increased support to encourage their participation in physical activities.

## Data availability statement

The raw data supporting the conclusions of this article will be made available by the authors, without undue reservation.

## Ethics statement

The studies involving humans were approved by Ethics Committee of the School of Physical Education of Hunan University of Science and Technology (No. ECSPEHNUST 2022/0012). The studies were conducted in accordance with the local legislation and institutional requirements. The participants provided their written informed consent to participate in this study.

## Author contributions

HW: Conceptualization, Funding acquisition, Investigation, Methodology, Writing – original draft, Writing – review & editing. XH: Writing – review & editing, Methodology, Writing – original draft, Writing – review & editing. YT: Investigation, Writing – original draft, Writing – review & editing. JT: Conceptualization, Investigation, Methodology, Project administration, Writing – original draft, Writing – review & editing. JY: Methodology, Supervision, Writing – original draft, Writing – review & editing.
